# 

**Published:** 1899-03

**Authors:** 


					﻿Fifty-Fourth Year,
VOL. L1V.
Whole Number,
DCXXVIII.
MARCH, 1899.
New Series.
VOL. XXXVIII.
No. 8.
BUFFALO
MEDICALJOURNAL
Established 1845 by AUSTIN FLINT, M. D.
EDITORS :
THOS. LOTHROP, M. D.
WM. WARREN POTTER, M. D.
ASSOCIATE EDITORS:
WILLIAM C. KRAUSS, M. D.
JOHN A. MILLER, Ph. D.
ERNEST WENDE, M. D.
JAMES WRIGHT PUTNAM, M. D.
JOHN PARMENTER, M. D.
ALVIN A. HUBBELL, M. D.
EUROPEAN AGENCY, J. B. BaILLHSRE A FILS. 19 Rue Hautefeullle, Pans.
Subscription, if paid, in advance, $2.00 ; otherwise, $2.50. Foreign subscription, $2.50.
Copyright 1899, by William Warren Potter, M. D.
Entered at the Post Office at Buffalo, N. Y., as second-class mail matter.
A, T. BROWN PRINTING HOUSE, BUFFALO. N. Y.
Table of Contents on Pajje V.
				

